# Neoantigens and NK Cells: “Trick or Treat” the Cancers?

**DOI:** 10.3389/fimmu.2022.931862

**Published:** 2022-07-07

**Authors:** Dan Lv, Muhammad Babar Khawar, Zhengyan Liang, Yu Gao, Haibo Sun

**Affiliations:** ^1^ Institute of Translational Medicine, Medical College, Yangzhou University, Yangzhou, China; ^2^ School of Life Sciences, Anqing Normal University, Anqing, China; ^3^ Jiangsu Key Laboratory of Experimental & Translational Non-Coding RNA Research Yangzhou, Yangzhou, China; ^4^ Applied Molecular Biology and Biomedicine Lab, Department of Zoology, University of Narowal, Narowal, Pakistan

**Keywords:** neoantigen, T cell, NK cell, cancer vaccine, adoptive T cell therapy

## Abstract

Immunotherapy has become an important treatment strategy for cancer patients nowadays. Targeting cancer neoantigens presented by major histocompatibility complex (MHC) molecules, which emerge as a result of non-synonymous somatic mutations with high immunogenicity, is one of the most promising cancer immunotherapy strategies. Currently, several therapeutic options based on the personalized or shared neoantigens have been developed, including neoantigen vaccine and adoptive T-cell therapy, both of which are now being tested in clinical trials for various malignancies. The goal of this review is to outline the use of neoantigens as cancer therapy targets, with an emphasis on neoantigen identification, clinical usage of personalized neoantigen-based cancer therapy agents, and the development of off-the-shelf products based on shared neoantigens. In addition, we introduce and discuss the potential impact of the neoantigen–MHC complex on natural killer (NK) cell antitumor function, which could be a novel way to boost immune response-induced cytotoxicity against malignancies.

## Introduction

According to immune surveillance theory, the immune system’s job is to keep the bodily environment stable and free of malignancies by detecting and destroying “non-self” tumor cells (1, 2). These tumor cells, on the other hand, try to evade immune surveillance in several ways, such as immunologic sculpting during tumor formation (1, 3, 4). The purpose of cancer immunotherapy is to increase the activity of the patient’s immune system to fight against cancer cells by natural mechanisms (5, 6). Checkpoint inhibitors, adoptive T cells, cancer vaccines, and antibody-based therapies are among the most clinically investigated immunotherapies thus far. T cells and natural killer (NK) cells are two of the most essential effector cells for recognizing and destroying tumor cells. The positive signals provided by precise identification of tumor antigens and the negative signals presented by immunological checkpoints can both be used to determine tumor-specific T-cell activation (7). Similarly, the activation of NK cells also relies on the integration of activating and inhibiting signals (8). As a result, tampering with the balance by blocking negative signals and boosting positive signals for T cells and NK cells may be advantageous to patients with cancer. To date, there have been many reports on therapies that block T- and NK-cell inhibitory receptors such as the checkpoint molecules CTLA-4 or PD-1/PD-L1. However, several pieces of evidence show that these strategies only bring benefits to a limited number of tumor-bearing patients, and the majority of patients still experience disease progression (9–12). As a result, therapies that can safely and effectively enhance the function of tumor-specific cytotoxic lymphocytes are required. Cancer neoantigens, which emerge as a result of non-synonymous somatic mutations in tumor cells and can be displayed by the major histocompatibility complex (MHC) molecules on the cell surface, may serve as a primary target for tumor-specific immune cells (13, 14). Indeed, neoantigen-based cancer vaccines and neoantigen-specific adoptive T-cell treatment alone or combined with immune checkpoint blockade (ICB) have had some progress (15–17). In this review, we primarily discuss the possibility of using neoantigens as cancer therapy targets by triggering the antitumor function of T cells, mainly focusing on neoantigen identification, clinical usage of personalized neoantigen-based cancer therapy agents, and the development of off-the-shelf products based on shared neoantigens. In addition, we introduce and discuss the potential impact of the neoantigen–MHC complex on the antitumor function of NK cells, which could be a novel way to boost immune response-induced cytotoxicity against malignancies.

## Cancer Neoantigens and Their Role in Cancer Immunotherapy

Cancer neoantigens are non-autologous antigens arising from non-synonymous somatic mutations occurring in tumor cells and that have the potential to be recognized in the context of MHC by T cells (15). These mutations, mainly containing single-nucleotide variants, splice variants, mutational frameshifts, and gene fusions, can generate aberrant proteins as malignancies grow (18–21). Varying people and cancer types have different types and numbers of mutations, and more neoantigens are expected to be formed in tumors having more mutations, whereas fewer tend to be generated in tumors having fewer mutations. The ability of T cells to identify mutant peptides in human tumors has been demonstrated for more than 20 years (22, 23). While CD4+ T cells recognize neoepitopes shown by MHC II molecules, CD8+ T cells identify neoantigens in the context of MHC I molecules expressed by tumor cells, which triggers T-cell cytotoxicity and tumor cell killing (24). Evidence suggests that the frequency of neoepitope-specific CD8+ T cells in tumor-infiltrating lymphocytes (TILs), as well as the presentation of neoantigens by MHC I molecules and the load of neoantigens on the surface of tumor cells, has a positive relationship with prognosis in patients with solid tumors (20, 25, 26). Thus, approaches of boosting T-cell responses specifically against neoantigens could be greatly beneficial to cancer patients in terms of clinical outcomes.

**Figure 1:**
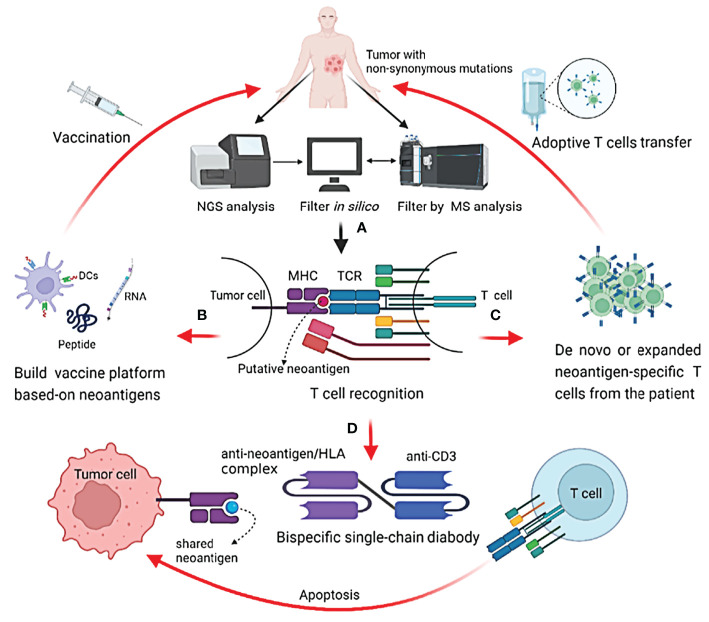
Identification of neoantigens and strategies to target them in cancer patients. **(A)** Identify potential neoantigens; **(B)** neoantigen-based cancer vaccine therapy; **(C)** neoantigen-specific T cell adoptive transfer; **(D)** mechanism of action of shared neoantigen-based bispecific diabody.

As for taking advantage of neoantigens in cancer immunotherapy, two main strategies have been employed in clinical trials (**Figure 1**). The first is T-cell adoptive treatment, which involves isolating immune cells from a patient’s tumor tissue and then injecting them back into the patient following *ex vivo* modification and amplification, primarily neoantigen-specific T cells (27). Another option is to design and produce a tailored vaccine against tumor cells that targets neoantigens to expand preexisting T-cell responses or induce new antitumor T-cell clones (28, 29). Some common mutations, such as TP53 and RAS family mutations, have been found in patients with the same or different types of malignancies (30–32). The possibility of off-the-shelf products based on neoantigens from these common mutations is being investigated, such as shared neoantigen-based vaccines and bispecific diabodies (**Figure 1**) (33, 34).

## Identification of Immunogenic Neoantigens

The development of next-generation sequencing (NGS) combined with *in silico* analysis in recent years has revolutionized the quick detection of neoantigens in cancer patients. To discover immunogenic neoantigens, the current framework often includes the following steps (**Figure 1**): obtaining patient tumor specimens and finding somatic mutations by NGS, predicting and selecting the possible neoantigens that can be presented by an MHC molecule of a given patient through *in silico* analysis or/and protein mass spectrometry (MS) analysis, and lastly testing the immunogenicity of the candidate neoantigens.

After NGS has revealed the mutations in clinical tumor specimens, computational techniques can be used to identify possible neoantigens. Whole-exome sequencing (WES) data of the cancerous and non-cancerous DNA can be used to map tumor-specific genetic abnormalities, and data from the RNA sequence can be compared with WES data to evaluate whether the mutant genes are expressed in tumors (35). To predict the binding affinity of the HLA (human leukocyte antigen, the MHC molecules in human) alleles with processed mutant peptides from cancer patients, several computational techniques have been developed, the majority of which rely on machine learning algorithms trained by extensive experimental datasets of HLA-binding peptides (21). NetMHCpan and NetMHCIIpan are two of the most often used algorithms for predicting peptide binding with HLA I and HLA II, respectively (36, 37). Meanwhile, MS analysis has been employed to scan the peptide repertoire shown on MHC I and II directly (38). *In silico* neoantigen predictions can be complemented with MS analysis of MHC-displayed peptide repertoire to limit possible neoantigens and increase prediction efficiency. Finally, the immunogenicity of predicted neoantigens should be determined experimentally, by testing their ability to induce T-cell activation, as not every mutant peptide displayed by MHC molecules can induce an immune response (39). The potential neoantigens can be employed in the development of immunotherapies. So far, several neoantigens have been discovered in preclinical and clinical investigations, some of which are derived from shared mutations in a range of malignancies (30, 31, 40–47) (**Table 1**).

**Table 1:** Potential shared neoantigens from common mutations in solid tumors.

Gene	AA mutation	Neoantigens	HLA restriction	Reference
*TP53*	p. R175H	HMTEVVR **H** C	HLA-A*02:01	(30, 44, 46)
	p. R248W	SSCMGGMN **W** R	HLA-A*68:01	(31)
	p. Y220C	VVP **C** EPPEV	HLA-A*02:01	
*KRAS*	p. G12D	VVVGA **D** GVGK	HLA-A*11:01	(41)
	p. G12V	VVVGA **V** GVGK	HLA-A*11:01	
	p. G12D	GA **D** GVGKSA(L)	HLA-C*08:02	(43)
	p. Q61L	ILDTAG **L** EEY	HLA-A*01:01	(45)
	p. G12V	VVVGA **V** GVGK	HLA-A*03:01	
*EGFR*	p. L858R	KITDFG **R** AK	HLA-A*11:01	(47)
	p. T790M	LTSTVQLI **M**	HLA-C*15:02	
*PIK3CA*	p. H1048R	EALEYFMKQMNDA **R** HGGWTTKMDWIFH	HLA-DRB1*04:05	(42)
*IDH1*	p. R132H	GWVKPIIIG **H** HAYGDQYRAT	HLA-DRB1*01:01	(40)

### Therapeutic Use of Personalized Neoantigens in Cancer Immunotherapy

Neoantigens are largely patient-specific due to each patient’s unique mutation repertoire. Autologous adoptive T-cell therapy and cancer vaccines based on individualized neoantigens are the two most common cancer immunotherapy methods studied in clinical trials and have indicated clinical advantages for solid tumors.

#### Adoptive T-Cell Therapy

Although tumor cells can be recognized and killed by TILs, adoptive transfer of T cells specific for certain neoantigens expressed by tumor cells may be more advantageous than an infusion of randomly isolated TILs after *ex vivo* amplification. The process of adoptive T-cell therapy (ACT) includes identifying neoantigens, expanding antitumor TILs *via* neoantigens, identifying or modifying neoantigen-reactive T cells, and lastly infusing the T cells back into the patient. Infusion of autologous neoantigen-reactive T cells in patients with a variety of solid tumors resulted in long-term regressions (48–54).

A phase II trial showed that 40 days after infusion of *ex vivo* amplified TILs that contained specific CD8+ T cells targeting neoepitope derived from the KRAS mutation G12D, a patient with metastatic colorectal cancer encountered regression of all the metastatic lung lesions, suggesting an important role of neoepitope-reactive CD8+ T cells in cancer therapy (51). Further research shows that TIL adoptive treatment is linked to an increase in neoepitope-specific CD8+ T cells (55). On the other hand, TIL-based ACT responders retained a subset of stem-like neoantigen-specific CD8+ T cells that show self-renewal and superior growth capacity *in vitro* and *in vivo*, highlighting the relevance of T-cell phenotypes in ACT response (56). In another case, after treating a widely metastatic cholangiocarcinoma patient with TILs containing Th1 cells specifically targeting neoantigens derived from the mutated erbb2 interacting protein (ERBB2IP), an obvious tumor size reduction was observed. Moreover, the size reduction of lesions in the lung and liver was shown again after retreatment with a pure population of neoepitope-reactive Th1 cells, suggesting the potential role of neoantigen-reactive CD4+ T cells in cancer treatment (50). Meanwhile, two HPV+ metastatic cervical carcinoma patients achieved total tumor regression that is ongoing for 44 months after adoptive transfer of TILs including neoantigen-targeted T cells and a relatively lower proportion of HPV-targeted T cells. This offered a new paradigm for immunotherapy of virally associated cancers: targeting neoantigens (52). Another clinical trial in a metastatic breast cancer patient found that adoptive transfer of TILs reactive against neoepitopes derived from four proteins combined with interleukin-2 and ICB resulted in complete durable regression for over 22 months (53), implying clinical benefits of combining ACT with other immunomodulators such as the checkpoint inhibitor. At the same, after adoptive transfer of enriched neoantigen-specific TILs combined with anti-PD-1 antibody pembrolizumab in a phase II pilot trial, three of the six patients with metastatic breast cancer showed objective tumor regression, including one complete response that was ongoing for more than 5.5 years (54).

In addition to *ex vivo* expanded antitumor T cells, ACT with modified T-cell receptor (TCR) or chimeric antigen receptor (CAR) has been demonstrated effective for cancer patients (57–59). One of the challenges for engineered T-cell therapies is to target tumor-specific antigens without destroying normal tissues (60, 61). Therefore, neoantigens may be a good target for engineered T-cell therapies. Recently, the molecular signatures of neoantigen-reactive antitumor T cells have been identified by single-cell RNA sequencing and TCR sequencing in a variety of solid tumors, and both neoepitope-targeted CD8+ and CD4+ T cells harbor distinct transcriptomic signatures compared with bystander T cells (62–66). This may provide new opportunities for cancer treatment by harnessing reprogrammed autologous T cells with enriched neoepitope specificities. In a recent report, a patient with progressive metastatic pancreatic cancer was treated with a single infusion of autologous T cells that had been genetically engineered to clonally express two allogeneic HLA-C*08:02-restricted TCRs targeting mutant KRAS G12D expressed by the tumors. This patient had regression of visceral metastases, and the response was ongoing at 6 months (67).

In general, using specific T cells to target neoantigens found only in tumor cells is a promising way to stimulate the activity of a patient’s immune system against cancer cells while minimizing the risk of toxicity, and many clinical trials are currently underway to investigate ACT as a monotherapy or in combination with checkpoint inhibitors (68).

#### Personalized Cancer Vaccines

Cancer vaccines utilizing tumor antigens have long been thought to be promising strategies for cancer treatment. Unlike traditional cancer therapeutic vaccines, which mainly focus on tumor-associated antigens (TAAs) that are abnormally expressed in tumor cells but can also be detected in normal tissues, neoantigen-based cancer vaccines have the capacity to amplify endogenous repertoire of T-cell responses specifically targeting neoantigen-expressed tumor cells, potentially reducing the risk of vaccine-induced tolerance or autoimmune responses (69). CD8+ T cells can be primed by antigen-presenting cells (APCs) expressing the neoantigen-MHC I complex after immunization, enhancing their cytotoxicity against neoantigen-expressing cancer cells. Dendritic cells (DCs, which are professional APCs), peptides, DNA, and RNA are the most common vaccination platforms. It is conceivable that immune responses induced by neoantigen-based vaccines could offer immunological memory and establish long-term protection against cancer recurrence. The viability and safety of tailored neoantigen vaccines have been demonstrated in human patients with solid tumors in clinical trials (70–76).

Three patients with stage III melanoma accepted an autologous DC vaccine including seven projected customized neoantigens as well as peptides from TAA-gp100 in a phase I trial (70). The vaccine can produce *de novo* T-cell responses while also increasing the response of preexisting neoantigen-reactive CD8+ T cells, indicating a broadening and diversification of T-cell responses (70). Similarly, 12 patients with advanced lung cancer received a tailored neoantigen-pulsed DC vaccine in another clinical trial. The disease control rate was 75%, and the median progression-free survival was 5.5 months. All treatment-related adverse events were grades 1–2, and there were no dose delays due to toxic effects (74). In another phase I trial, patients with high-risk melanoma were vaccinated with a lengthy peptide spanning 20 mutations each following the first curative-intent surgery. Following vaccination, neoantigen-reactive CD4+ and CD8+ T cells that had previously been undetectable were activated, with CD4+ T cells accounting for a larger frequency of the response. While four patients did not show disease recurrence for a median of 25 months after vaccination, two of the six patients who experienced recurrence a few months after vaccination were then treated with the anti-PD-1 antibody. Both of the patients showed complete clinical responses, highlighting the potential of combining ICBs and neoantigen therapeutic vaccines (69). Further research revealed that these patients developed memory T-cell responses with cytolytic capabilities *in vivo* that persist in the peripheral blood for years (75). In a phase Ib glioblastoma trial using a similar method with multi-epitope customized neoantigen peptide immunization, patients generated circulating neoantigen-specific T-cell responses, implying that neoantigen-targeting vaccines may have benefits in glioblastoma patients, which normally have a low mutation load (73). Meanwhile, the safety and function of neoantigen-based mRNA vaccines were tested in a study of patients with gastrointestinal cancer, although no clinical responses were observed in three of four individuals (77), while in another case, T-cell responses were elicited when patients with stage III/IV melanoma were given RNA vaccines that encoded neoantigens derived from specific mutations (10 per patient). Among the 13 patients, two had objective clinical responses, and one showed a complete response after combined with PD-1 blocking therapy (72).

According to these findings, tailored neoantigen vaccinations can trigger specific T-cell responses, and neoantigen-based vaccines in combination with ICBs could produce improved clinical results. Thus, personalized neoantigen-based vaccinations are being investigated in several clinical trials as monotherapy or in combination with checkpoint inhibitors (78).

### Therapeutic Strategies Based on Shared Neoantigens

Although personalized neoantigen discovery leads to attractive personalized therapeutics, high prices and time delays limit their use, and the challenges of predicting and identifying optimal neoantigens for each patient still remain. Despite each patient’s unique neoantigen repertoire, some neoepitopes can be found in various patients with the same or even in distinct forms of malignancy. Thus, ongoing research is being done to evaluate off-the-shelf anticancer therapeutics based on shared neoantigens, which could benefit multiple patients.

The mutation in isocitrate dehydrogenase 1 (IDH1) is common in diffuse glioma, and the most prevalent IDH1 mutation (R132H) generates a neoepitope presented by MHC class II molecules (40). In a phase I trial, 32 patients with IDH1 (R132H)+ astrocytomas were given an IDH1(R132H)-specific peptide vaccination and the vaccine-induced immune responses were observed in 93.3% of individuals, with vaccine-related adverse effects limited to grade 1. The progression-free rate after 2 years was 0.82 for patients who had immune responses (33). Similarly, immunogenic frameshift peptide (FSP) neoantigens resulting from mutations in coding microsatellites are shared by the majority of mismatch repair (MMR)-deficient malignancies, indicating that these FSP neoantigens may be utilized as targets to induce or amplify antitumor T-cell responses (79). Patients were subcutaneously vaccinated with shared FSP neoantigens (derived from mutant AIM2, HT001, and TAF1B genes) combined with montanide ISA-51 VG in a clinical phase I/IIa trial. All immunized patients showed immune responses to the vaccine. Three patients suffered grade 2 local injection site responses, but no vaccine-related serious adverse events happened (80). More preclinical and clinical investigations are being conducted to determine the effectiveness of shared neoantigen-based vaccines (**Table 2**).

**Table 2:** List of clinical trials employing off-the-shelf neoantigen vaccines.

Strategy	Tumor type	Drugs	Phase	ClinicalTrials. *gov identifier*
Vaccine	Melanoma	A vaccine made of 6MHP and NeoAg-mBRAF (a peptide BRAF585-614-V600E)	Phase I/II	NCT04364230
Vaccine	KRAS-mutated pancreatic ductal adenocarcinoma and other solid tumors	ELI-002 2P (Amph-modified KRAS peptides, Amph-G12D and Amph-G12R admixed with admixed Amph-CpG-7909)	Phase I	NCT04853017
Vaccine with checkpoint inhibitor	Intrinsic pontine glioma, intrinsic midline glioma (H3 K27M-mutant)	rHSC-DIPGVax vaccine Balstilimab Zalifrelimab	Phase I	NCT04943848
Vaccine with checkpoint inhibitor	Lung cancer, colorectal cancer, pancreatic cancer, other shared neoantigen-positive solid tumors	GRT-C903/GRT-R904 (shared neo antigen- based vaccine) Nivolumab Ipilimumab	Phase I/II	NCT03953235
Vaccine with checkpoint inhibitor	Malignant glioma	IDH1R132H peptide vaccine Avelumab	Phase I	NCT03893903
Vaccine with checkpoint inhibitor	Unresectable or metastatic deficient mismatch repair (dMMR) or MSI-H colorectal cancer, gastric or gastro-esophageal junction tumors	GAd-209-FSP MVA-209-FSP Pembrolizumab	Phase I/II	NCT04041310

In addition to neoantigen-based off-the-shelf vaccines, other strategies for the treatment of patients with common mutations are being tested, such as TCR-mimic antibodies, a type of agents that target the peptide–HLA complex in tumor cells (81, 82).

RAS oncogene mutations are found in different types of malignancies, and neoepitopes from RAS mutations are shared by several patients. Single-chain variable fragments specific for KRAS G12V mutation and NRAS Q61L mutation-generated peptide–HLA complexes were identified *via* phage display and transformed to bispecific single-chain diabodies (scDbs). *In vitro*, the scDbs were able to activate T cells to kill target cancer cells that expressed low endogenous amounts of the KRAS G12V or NRAS Q61L neoepitope–HLA complexes. In the mouse model, the scDbs employed *in vivo* similarly demonstrated a capacity to decrease the growth of tumors with KRAS G12V or NRAS Q61L mutation (45). Similarly, a bispecific scDb targeting the neoantigen produced from the R175H mutation of TP53, one of the most commonly mutated cancer driver genes, was developed and demonstrated an excellent affinity for the R175H mutant-derived neoepitope–HLA complex. Despite the low expression of TP53 R175H-derived neoantigen–HLA complex on the cancer cell surface, T cells were successfully activated to kill the cancer cells by the scDb *in vitro* as well as in mouse models (46). Recently, a TCR-mimic monoclonal antibody that could target a range of phospho-neoantigens displayed by HLA-A*02:01 in various tumor cells has been generated and has shown the capacity to induce T cells to kill tumor cells. The phospho-peptides derived from dysregulated protein phosphorylation in different types of tumor cells may serve as shared tumor-specific neoantigens (83). Based on these findings, a TCR-mimic antibody that can selectively target shared neoantigens while boosting T-cell function may theoretically be utilized to target malignancies with common mutations and could work as an off-the-shelf agent in cancer immunotherapy, although more research is required to testify this idea.

### The Possible Transformation of NK Cells’ Antitumor Function Based on Neoantigens

Human NK cells are the first line of antitumor lymphocytes, and lower NK-cell cytolytic activity has been linked to a higher tumor incidence (84–86). NK cells are cytotoxic toward tumor cells without prior activation and can regulate various immune responses by secreting immune-regulatory cytokines as well as chemokines (87–89). The combination of activating and inhibiting signals modulates the antitumor action of NK cells. Killer cell immunoglobulin-like receptors (KIRs) and natural killer group 2 A (NKG2A) are the two primary inhibitory receptors, both of which recognize HLA molecules (90). KIRs are transmembrane receptors of type I that majorly detect classical HLA I, which include HLA-A, HLA-B, and HLA-C (91, 92). KIR genes have several alleles, and variability within each gene allows the complex KIR repertoire to recognize changes in HLA I expression, which is itself highly polymorphic (93). KIRs with long intra-plasmatic tails and immunoreceptor tyrosine-based inhibitory motifs (ITIMs), such as KIR2DL1, KIR2DL2, and KIR2DL3, can interact with HLA-C, while others, including KID3DL1 and KIR3DL2, engage with HLA-A and -B (94). NKG2A, which forms a heterodimer with CD94, is a type II transmembrane receptor that can interact with a non-classic HLA molecule HLA-E (95).

**Figure 2:**
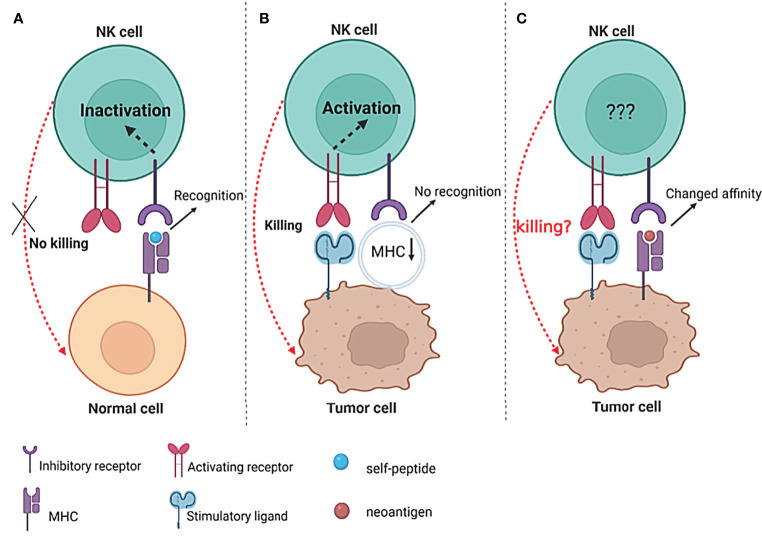
The possible influence of neoantigens on NK-cell function. **(A)** The interactions between inhibitory receptors and their specific MHC/self-peptide ligands inactivate NK cells, thus preventing cytolytic activity against healthy cells; **(B)** decrease or even lose the expression of MHC I on the surface of tumors, resulting in the “missing-self “recognition of NK cells to kill tumor cells; **(C)** the neoantigen presented by MHC I molecules on the surface of tumor cells may change the interaction affinity of MHC I and inhibitory receptors (such as KIRs) and finally influence the activity of NK cells.

Unlike T cells that detect the peptide in an MHC-restricted manner, NK-cell receptors that can recognize MHC molecules tend to bind to MHC itself and may be less specific for the provided peptide. However, the peptide could modify the interaction affinity of MHC with NK receptors (96). KIR3DL2 can interact with HLA-A3 and HLA-A11, and the interaction affinity appears to be highly dependent on the peptide displayed by HLA, with residue 8 playing a key role in recognition (97). Similarly, the interaction of KIR2DL2/3 with HLA-C was peptide selective. The bound peptide, particularly residues 7 and 8, can increase or abrogate the binding specificity of KIR2DL2/3 to HLA-C (98). Furthermore, peptides displayed by HLA I can operate as changed peptide ligands and effectively diminish KIR-mediated inhibition, indicating that alterations in the peptide presented by HLA I can influence NK-cell function (99). A peptide deriving from the core protein of hepatitis C virus (HCV) presented on HLA-C*03:04 modulates the function of NK cells by engaging the inhibitory receptor KIR2DL3 in a sequence-dependent manner, further implying that the binding of KIRs with HLA molecules and the function of NK cells can be influenced by the peptide (100). This specific peptide-dependent binding of KIR with HLA provides a potential mechanism for pathogens and self-peptides to influence NK-cell activation by varying inhibitory levels. The creation of wholly unique regions of amino acid sequence that can bind to MHC molecules should be conceivable as a result of the DNA changes accumulated in tumor cells. Following the malignant transformation of cells, the repertoire of peptides displayed by MHC molecules changes (13). Based on the fact that HLA–KIR interaction affinity is peptide-dependent and can influence the effector function of NK cells (97, 99–101), the altered peptide repertoire displayed by MHC molecules in cancer cells may be considered to influence the function of NK cells. As a result, it would be interesting to see if the neoantigens displayed by MHC molecules on tumor cells, especially for those that do not induce the downregulation of MHC I, may change the binding affinity of KIR-MHC and finally modify NK-cell activation (**Figure 2**).

The synergistic function of many NK-cell surface receptors determines the status of NK cells. Tumor cells can be rendered “invisible” to NK cells by upregulating inhibitory signals or/and downregulating activating signals on NK cells. The function of NK cells may even be modified by alteration in the affinity of the KIR–MHC interaction through the diverse peptide repertoire given by MHC molecules. Interfering with the activating and inhibitory signals has been utilized in several therapeutic techniques to boost NK-cell function (102–104). However, the possibly changed interaction affinity of the KIR-peptide/MHC complex resulting from the varied repertoire of a peptide given by MHC molecules in tumor cells may require more research.

### Perspectives

So far, strategies to boost the antitumor function of T and NK cells have yielded encouraging results. As knowledge of the neoantigens presented by MHC molecules expands, the research and clinical implementation of neoantigen-based therapeutic methods, including adoptive T-cell therapy and cancer vaccines, are full of potential in the clinical applications. However, there are still many questions required to be answered. For example, how may neoantigens should be identified and therapeutic strategies developed for cancers with modest mutation loads? Even though the discovery of neoantigen has the potential to lead to such appealing tailored treatments, high prices and time constraints limit their use. Thus, how can neoantigen-based therapeutics be developed faster and at a lower cost? Meanwhile, while neoantigen-targeted therapy for cancer patients is based on each patient’s unique neoantigen repertoire, the obstacles to predicting and selecting the best neoantigens for each patient remain. Because of that, can we better investigate some off-the-shelf cancer therapies based on common neoantigens and HLA allotypes? Furthermore, because neoantigen-based vaccines and adoptive T-cell treatment may be limited in their ability to overcome immune suppression caused by regulatory cells or tumor-derived factors as a monotherapy, they might be combined with other therapies to fully utilize their potential. Many studies indicated that the immune response can be boosted by conventional radiotherapies, chemotherapies, and targeted therapies (105–107). It remains to be determined, however, how and when combination therapy should be applied. Finally, now that intriguing customized treatment options based on neoantigen-reactive T cells have been brought, may new therapeutic approaches also be developed in NK cells against malignancies with a distinct peptide–MHC complex repertoire? All in all, a better understanding of the mechanisms underlying the activation of immune cells against cancer cells using neoantigens will undoubtedly aid the development of effective new mono- or combination cancer therapeutic strategies.

## Author Contributions

DL collected the data, drew the figures, and wrote the manuscript. HS proposed the idea and modified, supervised, and approved the final version of the manuscript. MK helped to edit the manuscript. ZL and YG provided professional pieces of advice in the minor revision previously. All authors contributed to the article and approved the submitted version.

## Funding

This study was funded in part by the Startup Foundation for Advanced Talents and Science and Technology Innovation Foundation at Yangzhou University (HS) and the Innovative Training Grant of College Students in Jiangsu Province (202111117097Y, YG, and HS).

## Conflict of Interest

The authors declare that the research was conducted in the absence of any commercial or financial relationships that could be construed as a potential conflict of interest.

## Publisher’s Note

All claims expressed in this article are solely those of the authors and do not necessarily represent those of their affiliated organizations, or those of the publisher, the editors and the reviewers. Any product that may be evaluated in this article, or claim that may be made by its manufacturer, is not guaranteed or endorsed by the publisher.
